# Development and validation of an objective assessment scale for chest tube insertion under ‘direct’ and ‘indirect’ rating

**DOI:** 10.1186/s12909-018-1430-9

**Published:** 2018-12-27

**Authors:** Ober Julian, Haubruck Patrick, Nickel Felix, Walker Tilman, Friedrich Mirco, Müller-Stich Beat-Peter, Schmidmaier Gerhard, Michael C. Tanner

**Affiliations:** 1 HTRG – Heidelberg Trauma Research Group, Center for Orthopedics, Trauma Surgery and Spinal Cord Injury, Trauma and Reconstructive Surgery, Heidelberg University Hospital, Schlierbacher Landstrasse 200a, D-69118 Heidelberg, Germany; 2 Department of General, Visceral and Transplantation Surgery Heidelberg University Hospital, D-69120 Heidelberg, Germany

**Keywords:** OSATS, Chest tube insertion, Education, Training, Video rating, Intermethod reliability, Interrater reliability, Construct validity, Direct rating, Indirect rating

## Abstract

**Background:**

There is an increasing need for objective and validated educational concepts. This holds especially true for surgical procedures like chest tube insertion (CTI). Thus, we developed an instrument for objectification of learning successes: the assessment scale based on Objective Structured Assessment of Technical Skill (OSATS) for chest tube insertion, which is evaluated in this study. Primary endpoint was the evaluation of intermethod reliability (IM). Secondary endpoints are ‘indirect’ interrater reliability (IR) and construct validity of the scale (CV).

**Methods:**

Every participant (*N* = 59) performed a CTI on a porcine thorax. Participants received three ratings (one ‘direct’ on site, two ‘indirect’ via video rating). IM compares ‘direct’ with ‘indirect’ ratings. IR was assessed between ‘indirect’ ratings. CV was investigated by subgroup analysis based on prior experience in CTI for ‘direct’ and ‘indirect’ rating.

**Results:**

We included 59 medical students to our study. IM showed moderate conformity (‘direct’ vs. ‘indirect 1’ ICC = 0.735, 95% CI: 0.554–0.843; ‘direct’ vs. ‘indirect 2’ ICC = 0.722, 95% CI 0.533–0.835) and good conformity between ‘direct’ vs. ‘average indirect’ rating (ICC = 0.764, 95% CI: 0.6–0.86). IR showed good conformity (ICC = 0.84, 95% CI: 0.707–0.91). CV was proven between subgroups in ‘direct’ (*p* = 0.037) and ‘indirect’ rating (*p* = 0.013).

**Conclusion:**

Results for IM suggest equivalence for ‘direct’ and ‘indirect’ ratings, while both IR and CV was demonstrated in both rating methods. Thus, the assessment scale seems a reliable method for rating trainees’ performances ‘directly’ as well as ‘indirectly’. It may help to objectify and facilitate the assessment of training of chest tube insertion.

## Background

Due to various changes in medical practice over the last years, education of junior doctors and medical students has become more diverse and challenging [1–3]. The kind of education practiced over the last decades known as ‘see one, do one, teach one’ is no longer feasible nowadays [4]. On the one hand, this type of education is limited by an increasing shortage of workforce in hospitals. On the other hand, teaching novices with the help of real patients is not always possible in respect of patients’ safety. Dated methods of learning and teaching also show a lack of objectiveness in the assessment of learning success. This leads to an unsteady quality of education [5, 6]. For these reasons, there is an increasing need for efficient, resource-sparing and objective educational concepts which combine a constant, high level of education on the one hand and maximum patient safety on the other [7].

An instrument suitable for making students’ curriculum and learning success more objective is the Objective Structured Assessment of Technical Skill Tool (OSATS). This tool, developed in the 1990’s at Toronto University (Canada), is currently one of the most frequently used for teaching skills in medical practice [8–10]. When using OSATS, a medical procedure is divided into various important key steps essential for the success of the specific intervention. Hereafter, an expert rates the performance of the trainee during the training session based on those key steps via 5-point Likert scale [10]. The total score of all key steps at the end of the training enables a low cost, readily available and objective evaluation of trainee’s performance. The only requirement for OSATS is the presence of an expert to rate the trainee which is called ‘direct rating’ [11]. A possibility to avoid the presence of an expert is the ‘indirect rating’ of the trainee through videos made during the training session [12]. To deliver meaningful results, all assessments in medical education need evidence of validity [13]. Here, the emerging concept of construct validity summarizes prior concepts of face, content and criterion validity [13, 14]. Therefore, construct validity comprises all these aspects of validation [13]. Construct validity assesses to which extend a test is able to differentiate between good and bad performance. Hence, analysis of construct validity is highly recommended for newly developed scores and assessment scales [15]. So far, both the validity of OSATS used with ‘direct ratings’ as well as its use with ‘indirect ratings’ has only been validated for the training of laparoscopic interventions [9, 15–18].

Chest tube insertion is the gold standard intervention for the treatment of lung and thoracic wall injuries [19]. For patients suffering from tension pneumothorax or respiratory insufficiency due to a pneumo- or haemothorax, a chest tube offers a life-saving possibility for a fast and safe restoration of respiratory function. An insecure and incorrect use of chest tubes potentially causes injuries of neighboring structures like blood vessels, lung tissue, abdominal organs or even the heart, which might lead to fatal complications. To ensure a maximum success for chest tube insertion combined with the best possible patient safety in critical situations, fast and safe execution of chest tube insertion is essential, for all treating doctors in an emergency setting. This shows the need for standardized, effective training methods as well as objective and structured feedback for the trainee. Thus, we developed an assessment scale and scoring system based on OSATS for chest tube insertion according to Hutton et al. [20]. As mentioned above validation of assessment scales is an essential requirement to ensure they deliver meaningful results. Therefore, prior to its use in surgical residency and training programs, the presented scale for chest tube insertion needs to be further evaluated and validated. Hence, the purpose of this study was to assess primarily the reliability of our scale when used in ‘direct’ and ‘indirect’ rating. Therefore, we analyzed the ‘intermethod reliability’ [21]. Intermethod reliability describes the comparison of the total score given via ‘direct’ rating on site to the one given by the video raters ‘indirectly’. Secondary, we sought to analyze both the construct validity of the developed scale when used in ‘direct’ and ‘indirect’ rating and the interrater reliability between the two ‘indirect’ raters.

## Methods

### Course and setting

**Fig. 1:**
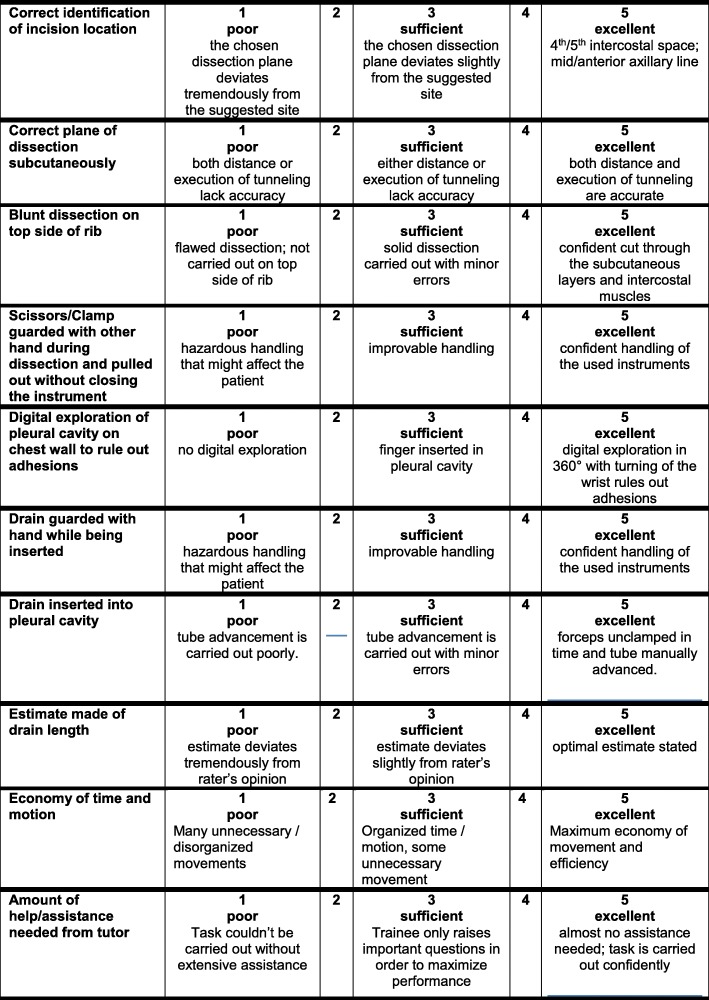
Scoring form for the developed assessment scale for chest tube insertion based on OSATS according to Friedrich et al. 2017. Reference: Friedrich M, Bergdolt C, Haubruck P, Bruckner T, Kowalewski KF, Muller-Stich BP, Tanner MC, Nickel F: App-based serious gaming for training of chest tube insertion: study protocol for a randomized controlled trial. *Trials* 2017, 18(1):56

This study was conducted between 04/2016–06/2016 at the Center for Orthopedics, Trauma Surgery and Spinal Cord Injury, Trauma and Reconstructive Surgery of Heidelberg University Hospital. For medical students, the course was offered as a voluntary training session for chest tube insertion as part of their regular surgical curriculum. All participating medical students (*N* = 59) were enrolled at the medical faculty of Heidelberg University during the time of their study participation. After a detailed theoretical introduction about chest tube insertion from the present expert rater, every participant carried out one chest tube insertion on a porcine thorax. In this exercise, one half of a porcine thorax (provided by a qualified and certified butcher) was laid out on a table poised on a soft foam pad. Students were presented with a chest tube and the necessary instruments for its insertion. Then they performed the insertion of a chest tube according to prior given instructions. Success or failure of insertion were easily controlled by examining the porcine specimen and therein positioning. This intervention was taped. The recorded image showed only the porcine thorax as well as the hands of the participants. The performance of every participant received a total of three ratings (one on-site in real-time, two via video) by three independent expert raters. All expert raters were attending surgeons recruited from the Center for Orthopedics, Trauma Surgery and Spinal Cord Injury, Trauma and Reconstructive Surgery Heidelberg University and had all 10 or more years’ experience in students’ education. During intervention, one present expert rated the participants (‘direct rating’). Later, every performance of the participants was rated by two other independent expert raters using the videos recorded during the trainings session (‘indirect rating’). All trainees were rated by the same three expert raters (one ‘direct’, two ‘indirect’). The rating of trainees was performed using our assessment scale for chest tube insertion [22] (Fig. 1). After the practical part, every participant received an individual questionnaire for self-evaluation. Trainees gave information about their individual training level as well as personal experience in using chest tubes.

### Developmental process of the assessment scale for chest tube insertion

Primarily, we conducted a review of the contemporary literature regarding existing scoring systems for chest tube insertion. We identified the ‘chest tube insertion scoring system’ developed by Hutton et al. in 2008 as a relevant and appropriate groundwork [20]. Hereafter, an interdisciplinary team of trauma and general surgeons were interviewed regarding key steps of correct chest tube insertion. In addition, the standard of care for chest tube insertion provided by the Heidelberg University Hospital was reviewed. In a final step, a team of experienced trauma and general surgeons that were also experienced lecturer evaluated all individual aspects and identified 10 key steps of correct chest tube insertion. Key steps were identified based on two factors. 1) safety, as most important and primary aspect; 2) ergonomics and speed, as secondary aspect. Each key step is scored from 1 (worst) to 5 (best), based on a 5-point Likert scale [22]. The maximum possible score was 50 points in total, the minimum score was 10 points.

### Validation

#### Primary endpoint

Primary endpoint was the analysis of the intermethod reliability when using the assessment sale for chest tube insertion in ‘direct’ and ‘indirect’ ratings. Therefore, in a first step the score given for trainees’ performance by the ‘direct’ rater was compared with the score given by ‘indirect rater 1’. In a second step the ‘direct’ score was compared to the score given by ‘indirect rater 2’. In a third step, we compared the score given via ‘direct’ rating on site with the average of the scores given by the two video raters ‘indirectly’.

#### Secondary endpoints

As secondary endpoints, we examined the interrater reliability as well as the construct validity of the scale. Ahmed et al. [23] defined the interrater reliability as the extent of conformity between ≥2 observers [23]. For analysis of the interrater reliability, the ‘indirect’ ratings via video records were compared. In a third step, construct validity when using the assessment scale in ‘direct’ and ‘indirect’ rating was analyzed [15, 23, 24]. The investigation of this question is based on subgroup analysis. Therefore, two subgroups based on the trainees’ self-evaluation regarding their experience in chest tube insertion were formed.

#### Ethics approval and consent to participate

Participation in the study was voluntary. The study was performed in concordance with the Declaration of Helsinki in its most recent form. Approval was received from the ethics commission of the University of Heidelberg (S-174/2016). Prior to study participation, every participant received information about the study. Informed consent to participate as well as written consent for anonymous data collection and anonymous recording of videos during the training sessions was obtained from each participant. All data were recorded anonymously, treated confidentially, and were evaluated by authorized staff for scientific purposes only. Participants’ names were kept separate from all study data and were not used for the study. Each participant was assigned a designated code that was used for the entire study documentation and data collection. All staff of the Heidelberg surgical center involved in the study was experienced in the handling of animal models and training devices used during the training session.

### Statistical analysis

Prior to statistical analysis, all data was completely anonymized. Data collection was carried out in MS Excel ® 2016 (Microsoft ®). Statistical analysis was performed via SPSS Statistics Version 24.0 (IBM ® Germany). For analysis of non-parametric related data, a Wilcoxon signed rank test was performed, for non-parametric, non-related data the Mann-Whitney U Test was carried out. Moreover, analysis of intermethod and interrater reliability was calculated by two-way random, absolute agreement calculation of the Intra Class Correlation Coefficient (ICC). Data is expressed as median ($\overset{\sim }{x}\Big)$ and interquartile ranges (I_50_). For all tests, a *p*-value less than 0.05 was considered significant. Graphical presentation of the results was done via box and whiskers-plots as well as bland-altman-plots.

## Results

### Participants

**Fig. 2:**
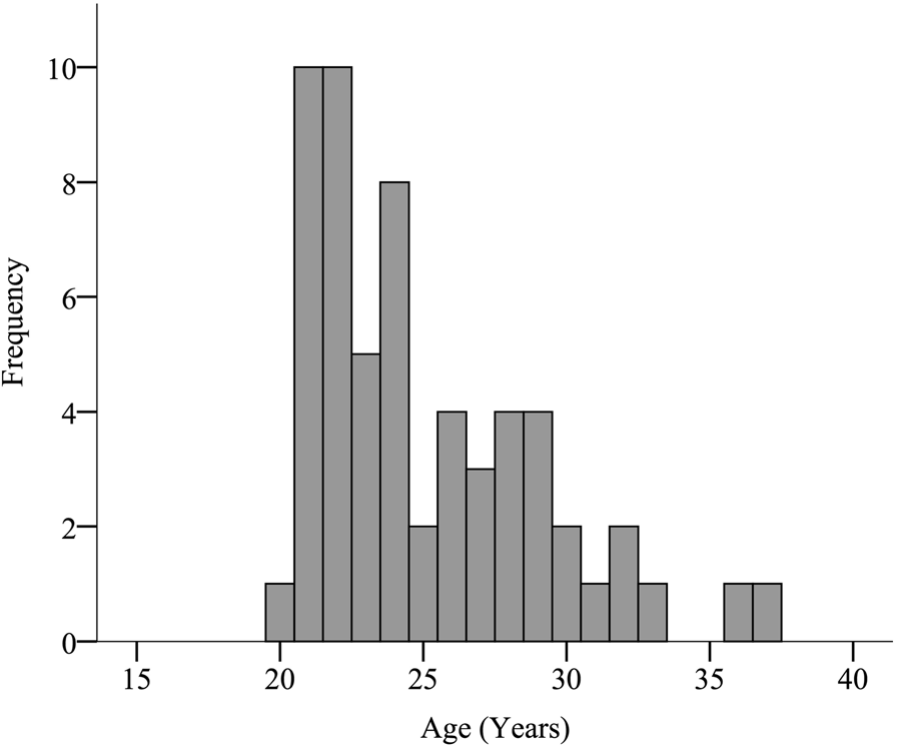
Age distribution among the participants. Here the age distribution among our collective of participants is presented by bar chart

We included 59 medical students to our study with no drop outs. Students’ median age was 24.0 (I_50_ = 6.0) (Fig. 2). At the time of study participation all students (*N* = 59) were at clinical study levels (3rd -6th year).

Prior to the study each participant stated the number of chest tube insertions seen outside the regular curriculum (e.g. in clinical traineeships during their holidays). For analysis of construct validity, every participant with ≥3 prior seen chest tube insertions was included in the ‘advanced’ group (*N* = 9). The range of prior chest tube insertions in the ‘advanced’ group was from 3 to 15. A ‘none’ group consisted of 9 randomly chosen participants that indicated that they had never seen a chest tube insertion before (Table 1).

**Table 1:** Subgroup characteristics

Subgroup	N	Median age (I_50_)	Number of seen chest tube insertions						
			0	1	2	3	4	10	15
Advanced	9 (50%)	28.0; 7.0	–	–	–	4	2	2	1
None	9 (50%)	26.0; 7.0	9	–	–	–	–	–	–

*N* = Number of participants; Average age in years is presented as mean and standard deviation

### Primary endpoint

#### Intermethod reliability

**Fig. 3:**
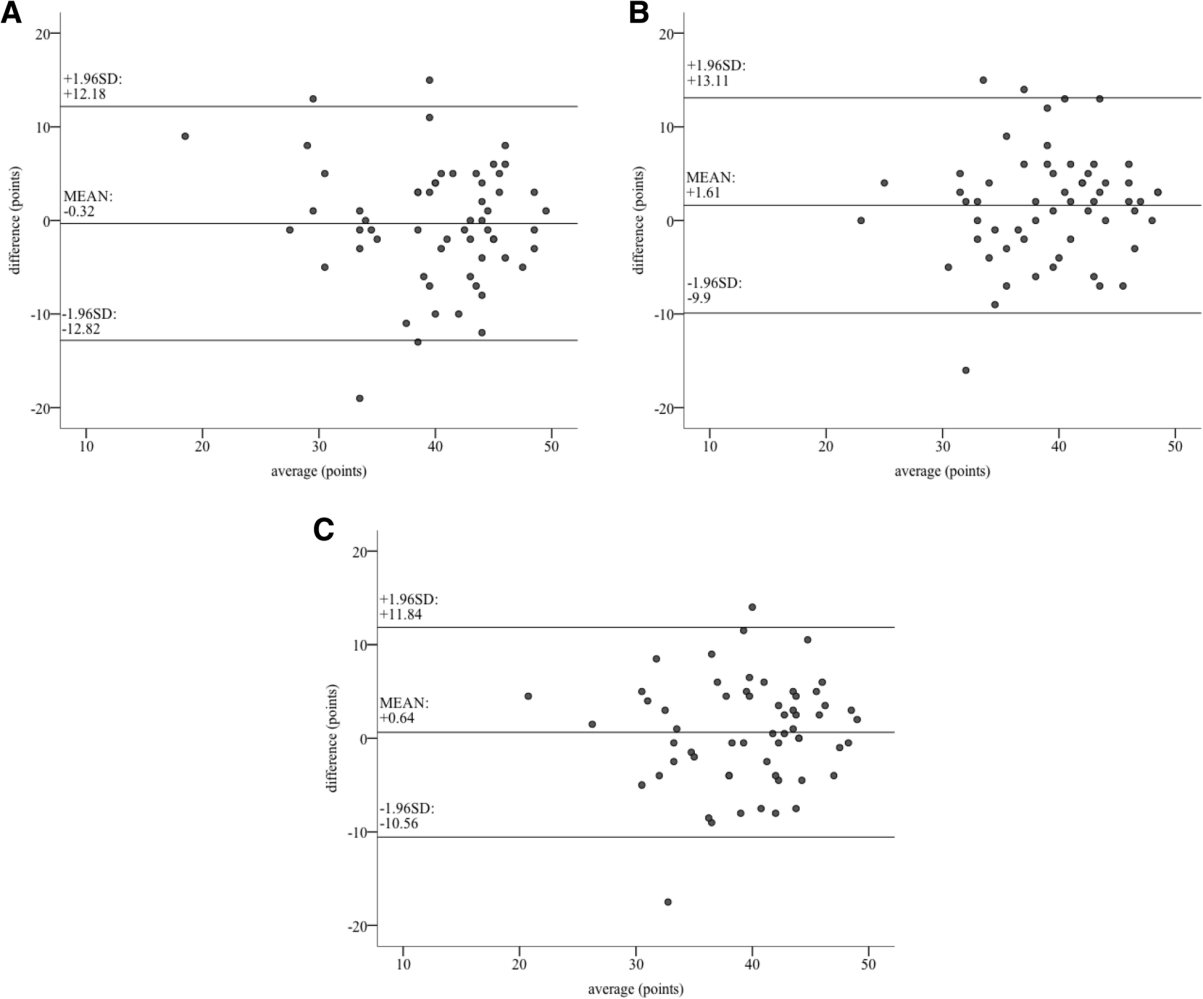
Visualization of the results for intermethod reliability. In this graph the results for intermethod reliability are visualized using bland-altman plots. In particular the scores given by the one-site rater are presented in context with **a** ‘indirect rater 1’, **b** ‘indirect rater 2’, **c** ‘average indirect ratings’. Here, the x-axis represents the average of given points, whereas the y-axis represents the difference between scores. SD = standard deviation

The median score given by the ‘direct’ rater was 41.0 points (I_50_ = 11.0). ‘Indirect rater 1’ rated the performance of the trainees better than the ‘direct’ rater did (42.0 points, I_50_ = 9.0). In contrast, the median score of ‘indirect rater 2’ was 39.0 points (I_50_ = 8.0). According to our data, the Wilcoxon test showed no significant difference between the ‘direct’ rating and the rating of ‘indirect rater 1’ (*p* = 0.851). Moreover Intra Class Correlation Coefficient (ICC) analysis showed a moderate agreement between the ‘direct’ and the ‘indirect rater 1’ (ICC = 0.735, 95% CI: 0.554–0.843) [25] (Fig. 3a). When comparing the score given in ‘direct’ rating to the score given by ‘indirect rater 2’ Wilcoxon test showed a significant difference between the scores given by those two raters (*p* = 0.041). The Intra Class Correlation Coefficient (ICC) analysis showed an ICC = 0.722 (95% CI 0.533–0.835) (Fig. 3b).

In a third step, we compared the ‘direct’ rating with the average score of the two ‘indirect’ raters (41.0 points, I_50_ = 9.5). According to our data, the Wilcoxon test showed no significant difference in between the ‘direct’ and average ‘indirect’ rating (*p* = 0.238). Intra Class Correlation Coefficient (ICC) analysis showed a good agreement between the ‘direct’ and the average ‘indirect’ rating (ICC = 0.764, 95% CI: 0.6–0.86) [25] (Fig. 3c).

### Secondary endpoints

#### Interrater reliability

**Fig. 4:**
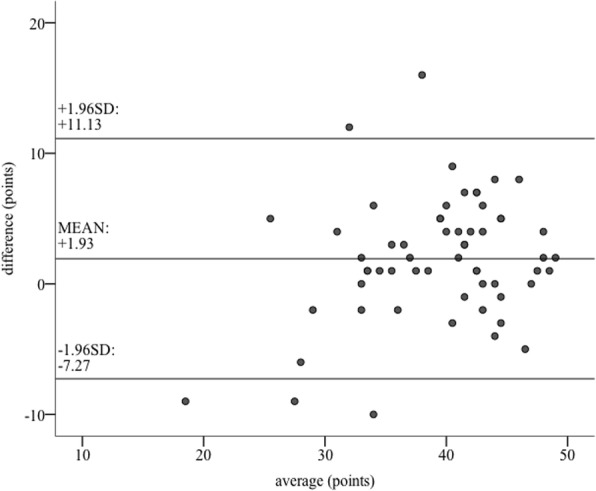
Visualization of the results for interrater reliability between the two ‘indirect’ ratings. In particular, data regarding interrater reliability is visualized using bland-altman plots. Here, the x-axis represents the average of given points, whereas the y-axis represents the difference between scores. SD = standard deviation

Results showed good correlation between the two ‘indirect’ raters (ICC = 0.84, 95% CI: 0.707–0.91) [25]. The median score given by the two ‘indirect raters’ differed significantly. (‘indirect rater 1’= 42.0 points, I_50_ = 9.0; ‘indirect rater 2’= 39.0 points, I_50_ = 8.0, *p* = 0.002) (Fig. 4).

#### Construct validity

**Fig. 5:**
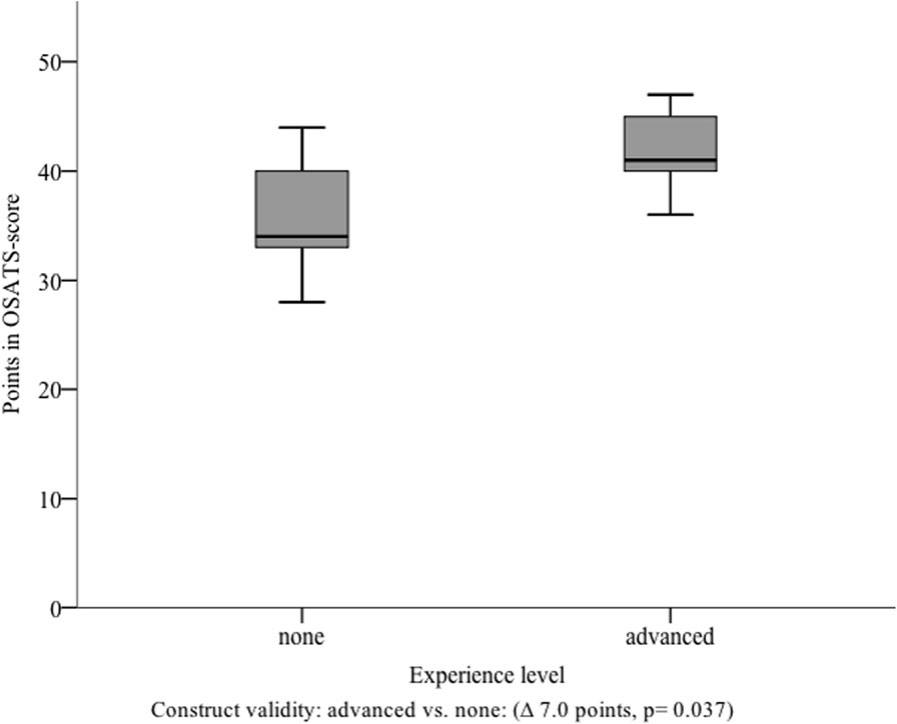
Results reached by the subgroups in ‘direct’ rating. Subgroup analysis showing the points reached for each group by ‘direct rating’, stratified by different experience levels; ∆ = difference

**Fig. 6:**
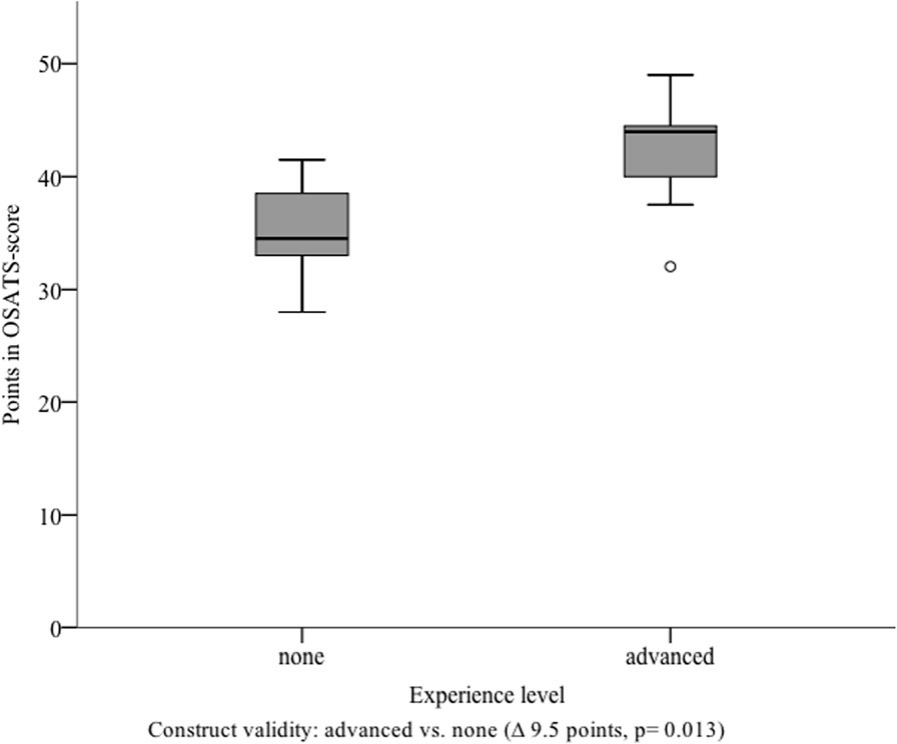
Results reached by the subgroups in ‘indirect’ rating Subgroup analysis showing the points given for each group by the two ‘indirect’ raters, stratified by different experience levels; ∆ = difference

The median score reached by the ‘advanced’- group in ‘direct’ rating (*N* = 9) was 41.0 points (I_50_ = 7.0). The ‘none’-group reached an average score of 34.0 points (I_50_ = 10.0). There was a significant difference between both groups (*p* = 0.037) (Fig. 5). When analyzing the results of the ‘indirect’ rating, the participants of the ‘advanced’ group (*N* = 9) reached a median score of 44.0 points (I_50_ = 7.3). The ‘none’ group (*N* = 9) reached 34.5 points (I_50_ = 7.5). For the difference between both groups Mann-Whitney U test also showed significance (*p* = 0.013) (Fig. 6). Based on those results, construct validity for our assessment scale, for ‘direct’ as well as for ‘indirect’ rating was proven.

## Discussion

Due to its ability to objectify and standardize expert ratings nowadays, OSATS scores are among the most frequently used scoring tools to evaluate trainees’ performance in medical practice [8–10]. Despite its popularity amongst medical lecturers no such score existed for teaching and scoring the placement of a chest tube. Thus, we developed an assessment scale and scoring system based on the OSATS to evaluate the performance of medical students during this procedure both ‘directly’ and ‘indirectly’. The purpose of the present study was to evaluate this score regarding its intermethod reliability when using it in ‘direct’ on site rating as well as in ‘indirect’ rating via videotaped interventions.

To analyze the intermethod reliability, we calculated the ICC between ‘direct’ and ‘indirect’ raters. Here, ICC values represent the conformity of scores that were given from different raters for each trainee, while placing a chest tube. Despite being an established tool for validation of assessment- tools, discrepancies remain regarding the interpretation of ICC values [12, 25–28]. In order to enhance interpretability of ICC values, we chose Koo et al.’s definition as they define a strict framework for interpreting ICC values. According to Koo et al., ICC analysis shows a moderate conformity for values between 0.5–0.75 and a good conformity for values between 0.75–0.9 [25]. The data from the current study indicated a moderate conformity between the ‘direct rater’ and both individual ‘indirect raters’. Interestingly, ICC analysis showed a good conformity when comparing ‘direct’ rating with the averaged ‘indirect’ ratings. While comparing the three independent ratings we found no significant differences between the ‘direct rater’ and ‘indirect rater 1’ or ‘averaged indirect ratings’. Surprisingly there was an unexpected significant difference between the score given by the ‘direct’ rater and ‘indirect rater 2’ (*p* = 0.041). It seems that ‘indirect rater 2′ was stricter in rating trainees’ performance than the other two raters. These differences might derive from an insufficient rater training. The importance of rater training for getting comparable results when using Global Rating Scales such as the OSATS is established [12, 15]. However, due to the substantial experience of all raters in both teaching medical students and performing this surgical procedure, rater training was kept reasonably short prior to commencement of the study. In retrospect, we believe that the conducted rater training was perhaps too short and additional rater training might lead to further improvement of the conformity between ratings. While this could explain varieties in the results obtained from different raters, we believe it does not implicate the validity of our developed assessment tool. Various studies investigating scoring systems for different applications have described a necessary reliability between ‘direct’ and ‘indirect’ ratings of being moderate or higher [15, 18, 26, 29]. Accordingly, the developed assessment tool enables sufficient conformity between ‘direct’ and ‘indirect’ raters. Considering our results, no superiority of one rating method could be proven. Therefore, based on these results we suggest to average the score of an on-site ‘direct’ rater and at least one ‘indirect’ rater. Even though the differences between ‘direct’ and ‘indirect’ ratings are small, it might be beneficial to use an average score of both rating methods in order to obtain the most accurate results. We believe with additional rater training the developed assessment scale has the potential to reduce the number of ‘indirect’ raters and might contribute to making the on-site rater abdicable.

As secondary endpoints, we examined the scale regarding its ‘indirect’ interrater reliability and its construct validity for ‘direct’ and ‘indirect’ rating. The ICC analysis for the interrater reliability proved good conformity between the two ‘indirect’ expert ratings, whereas comparison of the median revealed a significant difference between them (*p* = 0.002). The interrater reliability of the developed scale is higher than the one in other studies comparing two video-rater [12, 30], in particular Scott et al. [31] described a negative interrater reliability for their video raters. Furthermore, additional training of video raters potentially contributes to a further reduction of differences in scoring [12, 15].

The construct validity, which was evaluated in our study, includes aspects of structural, content and external factors. Therefore, it is considered as the most comprehensive aspect of validation of assessment scales such as the one here introduced [32]. Construct validity has already been proven for multiple OSATS scores of different specialist fields [15, 33–38]. Data of a small pilot study provided initial evidence regarding the construct validity of our developed scale. The results from the current study support these initial findings. Due to the fact that our study was offered as a part of the regular surgical curriculum all participants had completed the same previous courses resulting in a similar clinical experience. However, due to different clinical placements and voluntary extracurricular activities numbers of previously observed chest tube insertions varied. Thus, we used the number of prior seen chest tube insertions to build two groups with different experience levels. In our literature review we found no evidence for learning curves regarding the number of prior seen chest tube insertions. Nevertheless, the assumption that observation of surgical procedures leads to an improvement of surgical ability is in accordance with results of Schmitt et al., who showed skills improvement after observation of surgical procedures [39, 40]. Therefore, the cut-off for the ‘advanced’ group was set to ≥3 seen chest tube insertions as we expected that there were also learning effects concerning knowledge and precise conception of the intervention after several instances of seeing a chest tube insertion. Moreover, there was only a limited number of students who saw a chest tube insertion before study participation. Considering that, the cut-off of ≥3 resulted in an acceptable group size. According to our results, construct validity of our developed scale for chest tube insertion was shown for ‘direct’ as well as ‘indirect’ rating. In particular, it distinguished reliably between different experience levels of subgroups. Due to remaining differences between raters we believe that the developed assessment tool should be used for ‘formative’ assessments. Once, additional evidence regarding the validity of the developed score exists it has the potential to be integrated into ‘summative’ assessments.

## Limitations

Despite the positive results found for our developed scale, our study has limitations. It should be noted that we used only three independent expert raters in the current study. This design (one ‘direct’ and two ‘indirect’ expert raters) was chosen due to the applicability during the normal training curriculum. However, the lack of further raters, particularly ‘direct’ ones, limits the results of the current study. Further studies are needed including a higher number of raters in order to confirm our results. In addition, we were only able to prove construct validity after allocating medical students into different subgroups according to their level of experience based on the subjective self-evaluation of the participants regarding their previously seen chest tube insertions. It should be noted that this allocation of students into ‘none’ and ‘advanced’ groups by the count of prior seen chest tube insertions has no substantial supporting evidence due to missing literature and is therefore somewhat arbitrary. As a result, the results regarding construct validity should be interpreted carefully. However, our results support the evidence regarding construct validity gathered in an initial pilot study. In addition, this may cause a recall bias for medical students self-reporting their prior experiences. Moreover, it is possible that there were inaccuracies between the subgroups due to under- or overestimation of the participants’ self-assessment. It should also be mentioned that the results are based on subgroup analyses with relatively small subgroups which may cause a type I error. Additionally, the number of seen chest tube insertions within the ‘advanced’ group was from 3 to 15 which is a rather wide range. Abovementioned points might influence the interpretation of the results found for construct validity.

### Conclusion

Data of the current study provides evidence regarding an intermethod reliability of the developed assessment scale. In addition, findings support an interrater reliability of the scale for its use in ‘indirect’ rating and support construct validity for both rating methods. Good conformity between ‘direct’ and ‘indirect’ ratings indicates the equivalence of both rating methods for the developed scale. Due to remaining differences between ‘direct’ and ‘indirect’ ratings, it might be beneficial to use an average score of both rating methods in order to obtain the most accurate result, whereas, additional rater training potentially decreases variances between raters. It is therefore currently solely to use the developed assessment scale for ‘formative’ assessments. After further validation, the scale could potentially be integrated into ‘summative’ assessments. In conclusion, the evaluated assessment scale promises as a reliable and feasible method for rating trainees’ operative performances in context of chest tube insertion in ‘direct’ and ‘indirect’ rating.

## Abbreviations

CTI: Chest tube insertion
CV: Construct validity
ICC: Intra class correlation coefficient
IM: Intermethod reliability
IR: Interrater reliability
OSATS: Objective Structured Assessment of Technical Skill
