# Correction to: Development and validation of an objective assessment scale for chest tube insertion under ‘direct’ and ‘indirect’ rating

**DOI:** 10.1186/s12909-019-1491-4

**Published:** 2019-02-20

**Authors:** Julian Ober, Patrick Haubruck, Felix Nickel, Tilman Walker, Mirco Friedrich, Beat-Peter Müller-Stich, Gerhard Schmidmaier, Michael C. Tanner

**Affiliations:** 1 HTRG – Heidelberg Trauma Research Group, Center for Orthopedics, Trauma Surgery and Spinal Cord Injury, Trauma and Reconstructive Surgery, Heidelberg University Hospital, Schlierbacher Landstrasse 200a, D-69118 Heidelberg, Germany; 2 Department of General, Visceral and Transplantation Surgery Heidelberg University Hospital, D-69120 Heidelberg, Germany

**Correction to: BMC Med Educ (2018) 18:320**

**https://doi.org/10.1186/s12909-018-1430-9**

Following publication of the original article [1], the author reported that the given name and family name of all authors were swapped. The details are as follows:

**Incorrect names in the original article:**

Ober Julian

Haubruck Patrick

Nickel Felix

Walker Tilman

Friedrich Mirco

Müller-Stich Beat-Peter

Schmidmaier Gerhard

Michael C. Tanner

**Correct names:**

Julian Ober

Patrick Haubruck

Felix Nickel

Tilman Walker

Mirco Friedrich

Beat-Peter Müller-Stich

Gerhard Schmidmaier

Michael C. Tanner

The original article has been corrected.

## References

[1] Julian O (2018). Development and validation of an objective assessment scale for chest tube insertion under ‘direct’ and ‘indirect’ rating. BMC Med Educ.

